# Conceptualization of category-oriented likelihood ratio: a useful tool for clinical diagnostic reasoning

**DOI:** 10.1186/1472-6920-11-94

**Published:** 2011-11-17

**Authors:** Hamideh Moosapour, Mohsin Raza, Mehdi Rambod, Akbar Soltani

**Affiliations:** 1 Evidence-Based Medicine & Critical Thinking Working Team, Endocrinology and Metabolism Research Institute, Tehran University of Medical Sciences, Tehran, IR Iran; 2 Section of Neurosciences and Ethics, Chemical Injuries Research Center, Baqiyatallah University of Medical Sciences, Tehran, IR Iran; 3 Los Angeles Biomedical Research Institute at Harbor-UCLA Medical Center, Torrance, CA, USA

## Abstract

**Background:**

In the diagnostic reasoning process medical students and novice physicians need to be made aware of the diagnostic values of the clinical findings (including history, signs, and symptoms) to make an appropriate diagnostic decision. Diagnostic reasoning has been understood in light of two paradigms on clinical reasoning: *problem solving *and *decision making*. They advocate the reasoning strategies used by expert physicians and the statistical models of reasoning, respectively. Evidence-based medicine (EBM) applies *decision theory *to the clinical diagnosis, which can be a challenging topic in medical education.

This theoretical article tries to compare evidence-based diagnosis with expert-based strategies in clinical diagnosis and also defines a novel concept *of category-oriented likelihood ratio (LR) *to propose a new model combining both aforementioned methods.

**Discussion:**

Evidence-based medicine advocates the use of quantitative evidence to estimate the probability of diseases more accurately and objectively; however, the published evidence for a given diagnosis cannot practically be utilized in primary care, especially if the patient is complaining of a nonspecific problem such as abdominal pain that could have a long list of differential diagnoses. In this case, expert physicians examine the key clinical findings that could differentiate between broader categories of diseases such as organic and non-organic disease categories to shorten the list of differential diagnoses. To approach nonspecific problems, not only do the experts revise the probability estimate of specific diseases, but also they revise the probability estimate of the *categories of diseases *by using the available clinical findings.

**Summary:**

To make this approach analytical and objective, we need to know how much more likely it is for a key clinical finding to be present in patients with one of the diseases of a specific category versus those with a disease not included in that category. In this paper, we call this value *category-oriented LR*.

## Background

Diagnostic reasoning is one of the most difficult clinical skills particularly in the primary care setting. History taking and physical examination form the foundations of diagnostic reasoning [1]. Taking an adequate history and physical examination that suggest possible differential diagnoses are very important, so that without an adequate history and physical examination, all the subsequent diagnostic investigations might be misleading [2]. Despite its importance, significant weaknesses in the diagnostic skills among medical students, residents, and practicing physicians have been consistently reported [3,4]. Studies have shown that the most common error in the diagnostic reasoning of medical students and novice physicians is the use of non-discriminatory clinical findings (i.e. signs and symptoms) to support a given clinical diagnosis [5,6].

Medical students are usually taught to take a thorough history and perform detailed physical examination for all patients, which is quite different from the strategies that expert physicians use to make diagnosis [7]. Expert physicians use some key clinical findings to make a diagnosis or to decrease the number of differential diagnoses in a relatively short period of time. An expert physician uses such discriminatory signs and symptoms subjectively, while students, novice physicians and clinical educators need to be objectively aware of the diagnostic value of these discriminatory signs and symptoms in order to differentiate different diseases. Although the physicians' intuitive clinical experience is usually the main source of learning about the diagnostic value of clinical findings, such unsystematic experience may not be as accurate as the information obtained from high-quality diagnostic studies [8].

Diagnostic reasoning has been understood in the light of two paradigms for psychological research on clinical reasoning: *problem solving *and *decision making*. The former describes and advocates the reasoning strategies used by expert physicians to improve the instruction of medical students; the latter, on the other hand, emphasizes the statistical models of reasoning. Evidence-based medicine (EBM) applies *decision theory *to the clinical diagnosis [9], which can be a challenging topic in medical education. This article aims at introducing the new concept for revising the probability estimates of a *whole category of diseases *(instead of *one specific disease*) which we call *"category-oriented likelihood ratio" (LR) *hereafter in *evidence based clinical diagnosis*. Also the potential application of *category-oriented LRs*, as a novel strategy for evidence-based clinical diagnosis, is proposed and discussed in this article.

## Discussion

### Evidence-Based clinical diagnosis and its limitations

In the following sections, we describe the use of LRs in revising the probability of diseases. In order to make a better understanding of the concept of disease-oriented LR and category-oriented LR in diagnostic reasoning, we first outline two clinical scenarios below. Then we will use these two clinical cases in the rest of this publication.

#### Clinical scenarios

***Case 1: ***A 24-year-old healthy woman presents to a primary care physician complaining of an increased urinary frequency and burning pain during urination. She has had two episodes of prior urinary tract infections (UTI), and this episode seems "just like the previous one". She is sexually active with one partner and uses condoms with spermicidal jelly. Her physician considers UTI as the most probable diagnosis for the patient's specific complaints. The physician asks for and she denies having fever, back pain, nausea, vomiting, vaginal discharge, and hematuria. Here, following questions can be considered for clinical diagnosis:

• What is the diagnostic value of the presence or absence of the clinical findings such as dysuria, frequency or self-diagnosis in the diagnosis of UTI?

• How can a clinical educator objectively teach the students about revising the probability estimate of a differential diagnosis?

***Case 2: ***A primary care physician is evaluating a 38-year-old woman who complains of generalized abdominal pain from 3 months ago. The physician knows the long list of differential diagnoses such as disorders of stomach function and/or gastritis, infectious diarrhea, appendicitis, acute cholecystitis, diaphragmatic hernia, ulcerative colitis, malignant neoplasm of kidney, etc. which can be potentially the cause of the patient's nonspecific problem. In this scenario, following questions can be worthy of consideration:

• How should the primary care physician approach this non-specific problem in his patient?

• What are the important questions in history and physical examination in approaching this patient with a non-specific problem?

• Which important findings should the physician seek for?

• How would the physician advance from such a non-specific presentation to the potential differential diagnoses and then, to the final diagnosis?

• How could a clinical educator objectively teach diagnostic reasoning to the medical students to approach similar cases?

### Use of LRs to revise the probability estimate of a given disease

Medical students and novice physicians may not be clear enough about the exact diagnostic values of various clinical findings for a given diagnosis. This might partly have its origin in the utilization of traditional medical literature by medical students as the sources of knowledge rather than evidence-based literature [10]. In traditional medical literature, the signs and symptoms of a disease are generally listed without their predictive power in making a diagnosis [8]. For example, according to the traditional medical, frequency and dysuria support the diagnosis of urinary tract infection (UTI); the literature, however, does not yield any information about the relative diagnostic value of these clinical findings for the diagnosis of UTI objectively; nor does the traditional medical literature provide any quantitative information to estimate the probability of uncomplicated UTI in the presence or absence of such clinical findings (i.e. dysuria and frequency).

Evidence-based diagnosis attempts to improve the clinical diagnosis by applying research-derived evidence to the diagnostic decision-making process. The *probabilistic reasoning *in evidence-based diagnosis conveys the presence of uncertainty in every clinical decision. Using *probabilistic reasoning*, a diagnosis cannot (and doesn't need to be) completely ruled in/out. Instead, the probability estimate needs to be revised accurately for a disease to decrease the uncertainty and to exactly determine a physician's position regarding a predefined diagnostic threshold [11]. From this point of view, diagnostic reasoning is the process of revising the probability of having a disease by using new clinical and para-clinical data. Bayes' theorem is the standard approach to revise probabilities [9]. By applying Bayes' theorem to the new data obtained from clinical or para-clinical tests, the previous estimation of a disease probability could be revised [12-14]. For instance, the probability of UTI in *case 1 *would be revised depending on the presence or absence of hematuria using an objective and user-friendly diagnostic tool, known as likelihood ratio (LR).

Likelihood ratios indicate how much more likely it is for a test or a clinical finding to give a positive or negative result in a patient versus an individual free of the disease. Likelihood ratios are calculated from diagnostic studies as follow [15,16]:

$$\text{The}\;\text{likelihood ratio}\;\text{for a}\;\text{positive}\;\text{test[LR( + )] = }\frac{sensitivity}{1-specificity}$$

$$\text{The}\;\text{likelihood ratio}\;\text{for a}\;\text{negative}\;\text{test[LR( - )] = }\frac{1-sensitivity}{specificity}$$

The more distant the LR from 1, the more valuable it is for making a diagnosis. As a rule of thumb, LR(+) > 10 or LR(-) < 0.1 generate large and often conclusive changes from pre- to post-test probability and the likelihood ratios of 5 to 10 and 0.1 to 0.2 generate moderate shifts in pre- to post-test probability [15,16].

Table 1 shows the diagnostic accuracy of clinical symptoms and signs in the prediction of uncomplicated UTI [17]. This table shows that self-diagnosis has the greatest diagnostic value, which is twice the diagnostic value of hematuria. By applying simple formulae or a nomogram, the LRs can revise the pre-test probability estimate into a post-test probability (Table 2), while the pre-test probability is either the known prevalence of a disease or a physician's subjective impression of the probability of the disease in a given patient [15,16]. For example, according to table 1, the post-test probability of uncomplicated UTI in *Case 1 *is equal to 81% (Table 2).

**Table 1:** Diagnostic accuracy of symptoms and signs in predicting urinary tract infection as measured by positive and negative likelihood ratio (LR)*

Symptom/Sign	Positive LR	Negative LR
Dysuria	1.5	0.5
Frequency	1.8	0.6
Hematuria	2	0.9
Fever	1.6	0.9
Flank Pain	1.1	0.9
Lower Abdominal Pain	1.1	0.9
Vaginal Discharge	0.3	3.1
Vaginal Irritation	0.2	2.7
Back Pain	1.6	0.8
Self-diagnosis	4	0.1
Vaginal Discharge on Physical Examination	0.7	1.1
Costovertebral Angle Tenderness	1.7	0.9
Dipstick Urinalysis^#^	4.2	0.3

* Values are derived from the study by Bent et al. [17]

^# ^A positive result was defined as leukocyte esterase positive or nitrite positive, a negative result was defined as both negative.

**Table 2:** Calculation of post-test probability using likelihood ratios

1.	Odds = $\frac{probability}{1-probability}$
2.	Post-test odds = pre-test odds × likelihood ratios
	For example, according to table 1, post-test probability of uncomplicated UTI in case1 can be calculated as:
	Pre-test odds = $\frac{0.12}{\left(1-0.12\right)}=0.13$
	Post-test odds = 0.13 × 1.5 × 1.8 × 3.1 × 4 = 4.35
	Post-test probability = $\frac{4.35}{\left(1+4.35\right)}=81\%$

It should be mentioned that several LRs can be used together sequentially to calculate a single estimate of post-test probability, if the different clinical findings are independent. However, if the clinical findings are not independent, it is wiser to use the one with the better LR. For example, from two dependent clinical findings in *Case 1*, i.e. vaginal discharge and vaginal irritation, we only use LR (-) for vaginal discharge because it has a better discriminatory value and more reliability compared to the vaginal irritation. In situations similar to *Case 1 *that present with specific problems, the physicians can first formulate the differential diagnoses such as UTI and vaginitis. Then LRs can inform the physician about the value of different clinical findings to estimate the probability of each differential diagnosis.

In spite of the above-mentioned UTI case, most patients referring to primary care physicians have presenting complaints of nonspecific problems such as abdominal pain (as in *Case 2*) that have potentially a long list of differential diagnoses. In these circumstances, physicians may not formulate an earlier list of specific diseases for differential diagnosis. Also, *disease-oriented LRs *cannot help formulate objectively the differential diagnosis list either. Therefore, the LR-based diagnostic approach is not in line with the expert physicians' usual approach to nonspecific clinical problems [18].

An evidence-based diagnostic approach has some theoretical and practical limitations. LRs in evidence-based literature are *disease-oriented *and can only be used to diagnose a given disease, i.e. to revise the probability estimate of a given differential diagnosis. To formulate and to further decrease the number of differential diagnoses in the case of nonspecific clinical problems, another objective tool is needed. The following sections introduce the *category-oriented LR*, as the new objective tool.

### Expert-based clinical diagnosis and their drawbacks

### Conceptualization of revising category probability estimates

Moving from a clinical presentation towards a final diagnosis is not a predetermined procedure. Psychological research on problem-solving paradigm shows that expert physicians use several alternative strategies in clinical diagnosis depending on their clinical experience and familiarity with the problem that is presented to them [19]. Three different diagnostic reasoning strategies available to learners include [19]:

A. pattern recognition

B. hypothetico-deductive

C. scheme-inductive problem solving

The first strategy, *pattern recognition*, is an intuitive, experience-based process where clinical decisions are made rapidly and almost autonomically by perceiving that clinical findings from a new patient resemble a previously learned illness script [9]. In the second strategy, *hypothetico-deductive problem solving*, when patients present with a medical problem, physicians begin by generating a list of specific medical conditions that could logically explain the patient's problems namely differential diagnoses. Most physicians keep 3-5 differential diagnoses in their mind at one time which include: more common, more serious if undiagnosed and/or untreated, and more responsive to treatment [20].

In the case of specific problems, early formulation of differential diagnoses is straightforward. However, when nonspecific problems such as abdominal pain, fatigue, anorexia, fever, and weakness are encountered as presenting complaints, formulating a short differential diagnosis list would be complicated and cumbersome. In these situations, it may be useful to first think about the problem in terms of how it might be classified or categorized by etiology or other associated features such as anatomy, pathophysiology, body system, or more generic disease categories such as infectious, metabolic, neoplastic or psychiatric diseases and so on. At this step, looking for key clinical findings that can differentiate between the broader categories of diseases can help the physician decrease the number of differential diagnoses. For example, in approaching to fever, the presence of bone pain and weight loss suggests category of malignancy, the presence of rash and arthritis suggests category of inflammatory diseases, and the history of exposure to febrile patients or recent traveling suggests category of infectious diseases [19].

Teaching the third strategy mentioned above to medical students should be guided by a *scheme*. Various schemes have been considered to reflect an organized knowledge structure for the purpose of learning as well as providing a structure for diagnostic reasoning. By following such schemes, the physician actually looks for key clinical findings that will help him distinguish between the categories of conditions at the branching points of the scheme. The presence or absence of these clinical findings leads to the adoption of one category and the exclusion of the others. After several branching points, when the number of diagnostic options has been considerably reduced, the deductive reasoning or pattern recognition may be utilized [19]. Throughout this diagnostic reasoning strategy, especially in approaching the nonspecific problems, not only do the physicians revise the probability estimate of single diseases, but also they mostly revise the probability estimate of the categories of diseases. For example, if a patient is complaining of fatigue, lack of fever is a key clinical finding that decreases the probability of infectious disease category or lack of dyspnea on exertion is a key clinical finding that decreases the probability of cardiac disease category.

**Figure 1:**
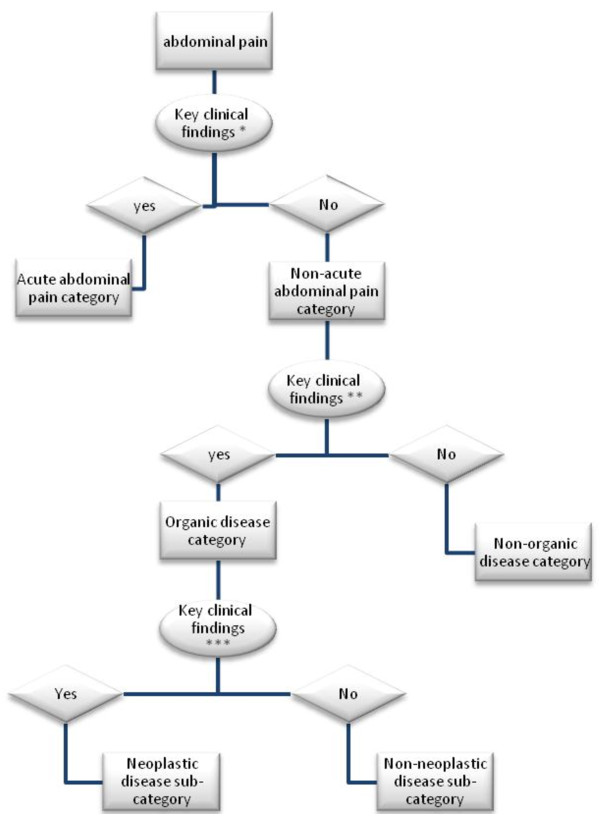
**An example for the scheme of an expert physician to approach abdominal pain^!^**. ^! ^This is an example for the experts' scheme of abdominal pain and not derived from a qualitative relevant study. * Onset of symptom. ** male gender, age > 60 years, history of blood in stool, pain affecting sleep, no pain relief after defecation, no specific character of pain, weight loss > 1 kg in 4 weeks. *** White blood cell count > 10,000 mm^-3^, erythrocyte sedimentation rate > 20 mm per hour, low hemoglobin level.

In *Case *2, an expert primary care physician is evaluating a 38-year-old woman with abdominal discomfort. The physician knows that the abdominal pain could be due to a disease from a long list of differential diagnoses including disorders of stomach function and/or gastritis, infectious diarrhea, appendicitis, acute cholecystitis, diaphragmatic hernia, ulcerative colitis, malignant neoplasm of kidney, etc [21]. The physician does not and cannot consider each of the diseases and use key clinical findings to distinguish them separately. However, before thinking about them separately and considering each of them as a differential diagnosis, a primary care physician would discern that the patient has acute or non-acute abdominal complaints and so he asks about the onset of problems (Figure 1). The patient says that her problem began almost 3-months ago. Considering that a non-acute abdominal complaint could be due to an organic or a non-organic disease, the physician considers evaluating the probability of the category of organic diseases in the patient. So he then asks for some clinical findings that have potential discriminatory power between the organic versus non-organic categories [21].

At this point, the physician asks about the history of blood in stool, pain affecting sleep, pain relief after defecation, the specific characteristic of the pain, and weight loss > 1 kg in 4 weeks. The patient mentions her pain relief after defecation and the fact that the pain does not affect her sleep. There is neither specific character to pain (such as description of the pain as one or more of the following: burning, cutting, terrible, feeling of pressure, dull, boring), nor significant weight loss nor history of blood in stool. At this point, non-organic category will be more probable and organic category less probable in the physician's mind.

The physician can again revise the differential diagnoses to evaluate the probability of a fatal sub-category of the organic category, i.e. neoplasm category. Hence, according to the key laboratory findings ordered for this purpose [21], the physician orders CBC and ESR for the patient. Normal WBC, hemoglobin and ESR convince the physician to treat the patient symptomatically, follow her up and hold further evaluation or refer to a specialist.

Therefore, by asking only few key questions and knowing the value of the discriminatory powers of such clinical findings, the physician can revise the probability estimates of the categories that include all pertaining diseases. In this step, the category of the disease is investigated.

At a critical point, these categorizations are not always mutually exclusive and most of these clinical findings may not be totally discriminative. Hence, the presence or absence of a clinical finding at a particular branching point can only increase or decrease the previous probability of those categories and does not rule in/out the diseases.

An important query which arises here is how to quantify the change in the probability of a category of diseases given a particular clinical sign or symptom. For example, in *Case 2*, the history of pain that does not affect sleep cannot totally rule in non-organic category or rule out organic category. However, like every other clinical finding, the pain that does not affect sleep can potentially decrease or increase the probability of a certain category. This example elucidates that expert physicians can subjectively revise the probability estimate of a disease category based on their past clinical experience, in the same fashion as they revise probabilities estimate of a disease. Medical students however may not easily understand and follow this approach. Therefore, clinical educators should explicitly teach the objective and quantified diagnostic value of these clinical findings to the medical students.

On the other hand, scheme-inductive problem solving and scheme trees serve as pathways for teaching, learning and clinical problem solving in some medical curricula [22]. However, these schemes and key clinical findings are mostly based on experts' personal experience and beliefs and may not be necessarily based on valid evidence. Expert physicians reach to the key clinical findings via trials and errors throughout several years of their clinical practice that may involve some degrees of cognitive biases [23,24]. Research on *disease-oriented *key clinical findings to revise the probability estimate of a given disease has shown that such clinical findings may be far less useful while considering their positive or negative likelihood ratios. For example, rebound tenderness, which is considered by many physicians as the diagnostic hallmark of acute appendicitis, has a positive LR of 1.9 [25]. This implies that a physician has to be more objective and vigilant while considering *category-oriented *key clinical findings as well as *disease-oriented *ones.

### Conceptualization of *Category-Oriented Likelihood Ratio*

A key question in dealing with patients presenting with non-specific complaints is whether a characteristic of the complaint or another sign/symptom can differentiate between two major categories of diseases. In the above scenario, the physician wants to know whether a pain that awakens the patient can discriminate between organic causes vs. non-organic etiologies. And if so, what the post-test probability of a certain category would be? While EBM emphasizes that key clinical findings for the diagnosis of a given disease are nothing but signs or symptoms with significant likelihood ratios (*disease-oriented LRs*) [26], the aforementioned questions will be answered by *novel concept of category-oriented LR *as defined in this article.

**Figure 2:**
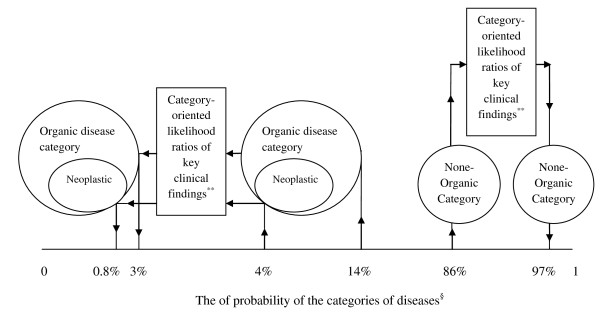
Revising the probability estimates of organic and non-organic disease categories using category-oriented likelihood ratios in approach to patients complaining of non-acute abdominal pain*. * Category-oriented likelihood ratios of the key clinical findings and the probabilities calculated using relevant values derived from the result of a study by Muris et al. [27]. ** Key clinical findings in this example are: male gender, age > 60 years, history of blood in stool, pain affecting sleep, no pain relief after defecation, no specific character of pain, weight loss > 1 kg in 4 weeks, white blood cell count > 10,000 mm-3, erythrocyte sedimentation rate > 20 mm per hour, low hemoglobin level. ^§ ^Arrows show the revising of the pre-test probability estimates to the post-test probability estimates.

Let's return to *Case 2 *and solve it objectively by *category-oriented LRs*. Regarding the prevalence, the pre-test probabilities of organic and non-organic categories are 14% and 86% respectively (Table 3) [27]. In order to revise the probability estimate of the organic category in approaching patients with non-acute abdominal pain, we have calculated *category-oriented LRs *for the most discriminatory clinical findings by using sensitivities and specificities from a study published by Muris et al. (Table 4) [27]. According to age and sex and after negative answers to 5 key discriminatory questions in the patient's history, the probability of organic category decreased to 4% and the probability of non-organic category increased to 96% (Table 5, Figure 2). Normal values of the 3 key discriminatory para-clinical findings again decreased the probability of organic to 3% and increased that of non-organic to 97%. Finally, the probability of neoplasm sub-category decreased using *category-oriented LRs *from 4% to almost 0.8% (Table 5).

**Table 3:** Final diagnoses in the patients with non-acute abdominal pain*

Final diagnosis	% of patients
**Non-organic**	
Abdominal symptoms (no diagnosis)	63.1
Disorders of stomach function/gastritis	7.6
irritable bowel syndrome	14.8
**Organic**	
infectious diarrhea, dysentery	0.4
other presumed infections	1.1
malignant neoplasm stomach	0.2
Malignant neoplasm colon, rectum	0.4
malignant neoplasm pancreas	0.2
malignant neoplasm other and unspecified sites	0.2
benign neoplasms (digestive tract)	0.9
Disease of oesophagus	0.4
Duodenal ulcer	1.7
other peptic ulcers	1.0
Appendicitis	0.1
inguinal hernia	0.1
hiatus (diaphragm) hernia	0.3
other abdominal hernia	0.1
diverticular diseases of intestines	1.4
chronic enteritis/ulcerative colitis	1.3
anal fissure/perianal abscess	0.4
cholecystitis/cholelithiasis	0.3
other disease digestive system	0.1
Haemorrhoids	0.6
malignant neoplasm trachea/bronchus/lung	0.2
pyelonephritis/pyelitis, acute	0.1
cystitis/other urinary infection	0.2
malignant neoplasm kidney	0.1
urinary calculus	0.4
other disease of urinary system	0.2
malignant neoplasm cervix	0.1
other malignant neoplasm (female genital system)	0.1
fibroid/myoma (uterus/cervix)	0.9
other diseases female genital tract	0.6

* Values are derived from the study by Muris et al. [27]

**Table 4:** Category-oriented likelihood ratios (LR) of signs and symptoms and laboratory results for organic diseases in patients with non-acute abdominal pain*

Patient Characteristics	Category-oriented LR(+)	Category-oriented LR(-)
Male sex	1.41	0.78
Age > 30 years	1.12	0.64
Age > 60 years	1.47	0.9
30 < Age < 60 years	1.08	0.88
**Symptoms**		
		
History of blood in stool	1.5	0.89
Pain affecting sleep	1.3	0.77
No pain relief after defecation	1.1	0.72
No specific character to pain^#^	1.5	0.93
Weight loss > 1 kg in4 weeks	1.29	0.89
**Laboratory tests**		
		
White blood cell count > 10000 mm^-3^	2.28	0.9
Erythrocyte sedimentation rate > 20 mm hour'	2	0.92
Low hemoglobin level	1.78	0.87

* Category-oriented LRs calculated using values of sensitivity and specificity derived from the study by Muris et al. [27]

^# ^No description of the pain as one or more of the following: burning, cutting, terrible, feeling of pressure, dull, boring

**Table 5:** Calculation of post-test probability of categories using *category-oriented likelihood ratios *in approaching non-acute abdominal pain

	According to table 3, post-test probability of organic diseases category in case 2 after negative answer about 5 key discriminatory questions in history taking can be calculated as:
1.	Pre-test odds = $\frac{0.14}{1-0.14}$ = 0.16
2.	Post-test odds = 0.16 × 0.78 × 0.89 × 0.77 × 0.72 × 0.89 × 0.93 = 0.05
3.	Post-test probability = $\frac{0.05}{1+0.05}$ = 4%
	Normal values of 3 key discriminatory clinical finding from table 4 again decrease the probability of organic category as:
1.	Post-test odds = 0.05 × 0.9 × 0.92 × 0.87 = 0.036
2.	Post-test probability = $\frac{0.036}{1+0.036}$ = 3%
	By regarding pre-test probability of neoplasm sub category, its probability can be revised as:
1.	Pre-test odds = $\frac{0.04}{1-0.04}$ = 0.041
2.	Post-test odds = 0.041 × 0.78 × 0.89 × 0.77 × 0.72 × 0.89 × 0.93 × 0.9 × 0.92 × 0.87 = 0.009
3.	Post-test probability = $\frac{0.009}{1+0.009}$ = 0.8%

This step generally deals with the 'category' of the disease rather than with the disease itself. To perform this task efficiently, the category-oriented LRs are inevitably needed. Therefore, by having *category-oriented LRs *and asking only few appropriate questions, the physician can objectively reduce the probability of a large number of differential diagnoses and limit the list of differential diagnosis.

## Summary

In this theoretical article, we attempted to compare and contrast evidence-based diagnosis and expert-based strategies in clinical diagnosis and proposed a new model combining both aforementioned methods. We also proposed the concept of *category-oriented LR *and suggested that the values of *category-oriented LRs *need to be calculated and tested in future studies. We believe that, not only can EBM properly use *disease-oriented LR*s to revise the probability estimate of given diseases, but it could also be helpful to revise the probability estimate of the category of diseases by using *category-oriented LRs*.

At this time, there is an increasing body of critically reviewed literature that emphasizes the diagnostic utility of specific physical findings [3,28]. Available systematic reviews present the accuracy of symptoms and signs in the diagnosis of different diseases in terms of sensitivity, specificity and LRs [29]. These systematic reviews also propose various diagnostic algorithms and/or scoring systems as diagnostic prediction rules that are based on the best available evidence [29]. Such tools are *disease-oriented*, too. Future studies are needed to calculate exact values for the *category-oriented LR*s of different clinical presentations in determining the various categories of diseases. We suggest the systematic and scientific empirical testing of experts' diagnostic schemes of different clinical presentations by *disease-oriented *and *category-oriented LRs *for objectivity and accuracy purposes. Similar to the evidence-based algorithms and prediction rules used for revising the disease probability estimates, future evidence-based diagnostic algorithms can be developed to revise the probability estimate of the category of diseases in the light of experts' schemes. So, increasing diagnostic evidence can gradually fall in line with today's clinical education which advocates the teaching of approaches to clinical presentations rather than teaching diseases within separate disciplines. The efficacy of clinical education and clinical practice using these evidence-based algorithms can be evaluated in future.

The virtue of EBM is becoming increasingly recognized in the medical community especially among primary care physicians [30]. We hope that physicians will soon begin to apply easily accessible and evidence-based *category-oriented LRs *in their practice to make diagnoses more rapidly and accurately.

## List of abbreviations used

EBM: Evidence-Based Medicine; LR: Likelihood Ratio: CBC: Complete Blood Count; ESR: Erythrocyte Sedimentation Rate; UTI: Urinary Tract Infection.

## Competing interests

The authors declare that they have no competing interests.

## Authors' contributions

HM had substantial contributions to concept and design, drafting the manuscript, revising it critically for important intellectual content and writing and final approval of the version to be published. MR had substantial contributions to concept and design, drafting the manuscript, revising it critically for important intellectual content and writing and final approval of the version to be published. MR had substantial contributions to concept and design, drafting the manuscript, revising it critically for important intellectual content and writing and final approval of the version to be published. AS developed the main concept and hypothesis, had substantial contributions to concept and design, drafting the manuscript, revising it critically for important intellectual content and writing and final approval of the manuscript.

## Authors' information

• Hamideh Moosapour (M.D., Student of medical education) research assistant, EBM & Critical Thinking Working Team, Endocrinology and Metabolism Research Center, Tehran University of Medical Sciences.

• Mohsin Raza (M.D., PhD) the founder and current director of the faculty development program at the Baqiyatallah University of Medical Sciences and associate professor of neuroscience, at the Section of Neurosciences and Ethics, Chemical Injuries Research Center, Baqiyatallah University of Medical Sciences

• Mehdi Rambod (MD), post-doctoral fellow at Los Angeles Biomedical Research Institute at Harbor-UCLA Medical Center, Torrance, CA, USA

• Akbar Soltani (M.D., M.A. in Philosophy, student of medical education) associate professor of endocrinology and the head of EBM & Critical Thinking Working Team, Endocrinology and Metabolism Research Center, Tehran University of Medical Sciences

## Pre-publication history

The pre-publication history for this paper can be accessed here:

http://www.biomedcentral.com/1472-6920/11/94/prepub
